# Is There a Real Relationship between the Presence of *Helicobacter pylori* in Dental Plaque and Gastric Infection? A Genotyping and Restriction Fragment Length Polymorphism Study on Patient Specimens with Dyspepsia in Southwest Iran

**DOI:** 10.1155/2023/1212009

**Published:** 2023-11-07

**Authors:** Mojtaba Moosavian, Elyas Kushki, Tahereh Navidifar, Eskandar Hajiani, Mahdi Mandegari

**Affiliations:** ^1^Infectious and Tropical Diseases Research Center, Health Research Institute, Ahvaz Jundishapur University of Medical Sciences, Ahvaz, Iran; ^2^Department of Microbiology, School of Medicine, Ahvaz Jundishapur University of Medical Sciences, Ahvaz, Iran; ^3^Division of Gastroenterology and Hepatology, Department of Internal Medicine, Ahvaz Jundishapur University of Medical Sciences, Ahvaz, Iran; ^4^School of Dentistry, Ahvaz Jundishapur University of Medical Sciences, Ahvaz, Iran

## Abstract

**Background:**

The oral cavity can act as an extra gastric reservoir for *H. pylori*, and the presence of the bacteria in the oral cavity is associated with a higher risk of dental caries development. This study aimed to determine the genotype and evaluate the association between the presence of *H. pylori* in dental plaque and gastric biopsy specimens in dyspeptic patients in Ahvaz, Southwest Iran.

**Methods:**

In this study, 106 patients with recruited dyspeptic complaints were selected, and from each patient, two gastric antral biopsy specimens and two dental plagues were examined. The presence of *H. pylori* was identified by the rapid urease test (RUT) and the amplification of *ureAB* and *16S rRNA* genes. Also, to verify a hypothetical mouth-to-stomach infection route, the enzymatic digestions of three genes of *cagA*, *vacA*, and *ureAB* in *H. pylori* strains isolated from dental plaques and stomach samples were compared for each same case.

**Results:**

*H. pylori* was found in the stomach of 52.8% (56/106) and the dental plaques of 17.9% (19/106) of the studied cases. On the other hand, *H. pylori* was recognized in the stomach of all 19 cases with oral colonization. Following a combination of restriction fragment lengths 21 polymorphism (RFLP) patterns of these three known genes on stomach and dental plague samples, 14 and 11 unique patterns were seen, respectively. However, for all *H. pylori*-positive cases (19), the comparison of RLFP patterns of these genes in dental plaque and gastric biopsy specimens was different for the same case.

**Conclusions:**

In this study, it seems that there is no significant association between the presence of *H. pylori* in dental plaque and the stomach of the same case.

## 1. Introduction

*Helicobacter (H.) pylori* has been known as one of the most common agents of human bacterial infections. This bacterium is located under the gastric mucous layer, adjacent to the gastric epithelial cells. Infection caused by this bacterium is now recognized as a serious transmissible infectious disease [1]. *H. pylori* infections are common in areas with low levels of social-economic health [2]. Despite many investigations, the exact route of infection transmission of this microorganism is still unknown. However, several different modes are suggested: gastro-oral, oral-oral, and fecal-oral transmission [3]. *H. pylori* is the only known pathogenic bacterium that survives in the highly acidic environment of the human stomach. This bacterium by producing a urease enzyme that converts urea into carbon dioxide and ammonia increases the pH and neutralizes the acid in the stomach environment. Reports also show that for colonization in the gastrointestinal mucosa, strains of *H. pylori* produce more urease enzymes than other pathogens [4, 5].

In addition to the stomach, *H. pylori* has also been isolated from the saliva and feces of the human as a likely reservoir of it [6]. Since the oral cavity can act as an additional gastric reservoir for *H. pylori*, it may lead to gastric reinfection and is, therefore, a possible factor for eradication failure and reinfection [7]. On the other hand, Moseeva et al. found a relationship between the colonization of this bacterium in the oral cavity and the risk of dental caries development [8]. In contrast, some reports did not show the simultaneous presence of *H. pylori* in the mouths of patients with gastric infection, because oral and stomach *H. pylori* had different genotypes [9, 10].

These controversial results may be due to several factors including the variations in the study population, sampling procedures, and methodologies used for the detection of *H. pylori* in dental plaques. There are several diagnostic tests for *H. pylori* in dental plaques, including rapid urease test (RUT), polymerase chain reaction (PCR), immunoassay, cytology, and bacterial culture. In general, the detection rate of *H. pylori* reported in studies was higher using RUT than other tests. The lowest detection rate was when microbial culture was used to detect the presence of this bacterium in dental plaque [11].

Several virulence factors have been identified in *H. pylori* that can develop the severity of pathogenicity. Among them, cytotoxin-associated gene A (*cagA*) and vacuolating cytotoxin A (*vacA*) have been mentioned as the two main virulence factors of *H. pylori* that are involved in the pathogenesis of different strains. The *cagA* gene is located within a 40-kilobase DNA fragment and is known as the cag pathogenicity island (*cag* PAI). The *cag* PAI encodes nearly 27 proteins, one of the most important of which is oncoprotein. Twenty of these proteins have been designed as cag type IV secretion system (T4SS), which can induce the production of interleukin 8, mucosal inflammation, and an increased risk of the peptic ulcer [12, 13]. Some reports showed that 48% and 80% of *H. pylori* strains isolated from Thai and Korean patients, respectively, had an intact *cag* PAI gene [14]. The *vacA* is a housekeeping gene that can induce vacuolation and apoptotic processes in epithelial cells, as well as immunosuppressive effects on some immunological cells [12].

In this study, to investigate the hypothesis of oral-to-stomach transmission in *H. pylori* infection, it was necessary to compare the RFLP patterns of several *H. pylori* genes between the mouth and stomach for the same case. This method was also able to analyze DNA extracted from *H. pylori* digested by high-frequency restriction endonucleases and showed distinct digestion patterns between strains [15]. In our study, three genes of *cagA*, *vacA*, and *ureAB* were selected for these analyses. To our knowledge, no sufficient research has been conducted on the detection of *H. pylori* in dental plaque and gastric biopsy specimens isolated from patients with dyspepsia in Ahvaz, Southwest Iran. 

Therefore, the purpose of this study with registration number OG-93129 was to determine the genotype and investigation of *Helicobacter pylori* in dental plaque and gastric biopsy samples, as well as evaluate the relationship between Helicobacter pylori colonization in dental plaque and stomach

## 2. Materials and Methods

### 2.1. Study Population and Sampling

In this study, specimens were collected from 106 patients with dyspeptic complaints who were referred to the teaching hospital of Imam Khomeini, Ahvaz, Iran. Initially, two biopsy specimens from each patient were taken by a gastroenterologist from the antral, within 5 cm of the pylorus. The exclusion criteria were as follows: treatment with any antibiotics, proton pump inhibitors, H2 blockers, or bismuth compounds during one month preceding this study, recent use of nonsteroidal anti-inflammatory drugs, periodontal therapy within the past year, and signs of severe periodontal infections within the preceding 6 months [16].

We retained one gastric biopsy specimen for RUT and stored the other at −70°C for DNA extraction. To perform RUT, samples were inoculated into the urea broth medium (Sigma Co.). The required time for a positive test depends on the concentration of *H. pylori* and temperature. Although most RUTs will change to positive within 120–180 minutes, for negative tests and increased sensitivity, it is best to read the test for 24 hours, so in this study, the results were interpreted after one overnight.

On the other hand, two dental plaque samples were taken from each patient before endoscopic examination. To remove the remaining foods, each patient was requested to wash his/her mouth with normal saline before sampling. Dental floss was used to completely clean the interdental spaces. The samples were transported in the tubes containing digestion buffer (100 mM NaCl, 10 mM Tris-HCl (pH 8.0), 250 mM ethylene diamine tetra acetic acid (EDTA) (pH 8.0), and 1% sodium lauryl sarcosine) on the day of sampling [6]. One of these samples was used for screening *H. pylori* by RUT, and the other was stored at −70°C for DNA extraction and PCR.

### 2.2. DNA Extraction

Samples were crushed and mixed by a vortex. Then, the mixtures were centrifuged at 12000 × *g* for 3 min. A volume of 200 *μ*l of suspended pellets in normal saline was transferred to a 1.5 ml microtube. The genomic DNA was extracted using a High Pure PCR Template Preparation Kit (Roche Diagnosis, Mannheim, Germany) according to the protocol of DNA extraction from mammalian tissues. The concentration of the extracted DNA was measured at 260 nm, using a NanoDrop instrument (Thermo Scientific, USA) and gel electrophoresis. DNA purity was acceptable when the light absorption ratio at 260 nm to 280 nm was between 1.5 and 2.

### 2.3. Identification of *H. pylori* Using PCR

*Helicobacter pylori* was identified by PCR amplification of *ureAB* (encoding urease enzyme conserved in all Helicobacter isolates), as well as *vacA*, *cagA*, and *16S rRNA* genes [17, 18].

The sequence of used primers is shown in Table 1. For each gene, a uniplex PCR reaction was prepared in a volume of 25 *μ*L. The master mixture consisted of 0.02 units of Taq DNA polymerase, 1.5 mM MgCl2, 0.4 *μ*M of each primer, 1xPCR buffer, 0.2 mM dNTP mix, 25 ng DNA template, and distilled water up to a final volume of 25 *μ*l.

The amplification reaction was carried out in Mastercycler Nexus Thermal Cycler Gradient (Eppendorf, Hamburg, Germany) with an initial denaturation step at 94°C for 5 min, followed by 35 cycles with the following profiles: for 16S rRNA, 94°C for 1 min, 59°C for 1 min, and 72°C for 1 min; for *vacA*, 94°C for 1 min, 58°C for 1 min, and 72°C for 1 min; for *cagA*; 94°C for 45s, 50°C for 45 sec, and 72°C for 45 s; and for *ureAB*, 94°C for 1 min, 50°C for 1 min, and 72°C for 2 min [17, 19].

After electrophoresis of PCR products and staining of 2% gel agarose with ethidium bromide, the amplicons were observed and photographed using an ultraviolet light gel documentation system (Viber, Germany).

### 2.4. Restriction Fragment Length Polymorphism (RFLP) Analysis

The *vacA*, *cagA*, and *ureAB* gene fragments were amplified by PCR and then were digested with HaeIII and Sau3AI for *ureAB*, HhaI and HphI for *vacA*, and HinfI for *cagA* for 3 hours at 37°C in the appropriate buffers suggested by the supplier (Fermentas, US). The digested products of each gene target were electrophoresed on 2% agarose gel stained with ethidium bromide [20].

### 2.5. Statistical Analysis

The descriptive statistics and chi-square test were performed in SPSS version 16.00, and the significance level of *p*  <  0.05 was used in this study.

## 3. Results

A total of 106 gastric biopsy and dental plaque specimens from patients with dyspeptic complaints were evaluated. Of these patients, the number of male cases (88) was significantly higher (*p*  <  0.05) compared with women (18) cases.

The patient's mean age was 33.9 ± 2.07 years. The patients were classified into four main groups based on clinical diagnosis including 58 cases with gastritis (54.7%), 35 cases with peptic ulcer (33%), 11 cases with duodenum ulcer (10.4%), and 2 cases of asymptomatic.

**Figure 1:**
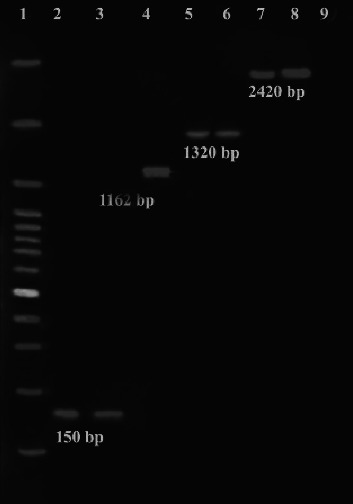
Lane 1: size marker of 100 bp; lanes 2 and 3: PCR products of *16S rRNA* (150 bp), lane 4: PCR product of *vacA* (1162 bp), lanes 5 and 6: PCR products of *cagA* (1320 bp), lanes 7 and 8: PCR products of *ureAB* (2420 bp).

In this study, the prevalence of *H. pylori* in the stomach specimens (56/106, 52.8%) compared with the dental plaques (19/106, 17.9%) specimens was significantly higher (*p*  <  0.05). On the other hand, *H. pylori* was found in the stomach of all 19 cases with dental plaque colonization. All patients with *H. pylori* colonization in dental plaque and gastric biopsy had positive results in both molecular identifications of *ureAB* and *16S rRNA* genes and RUT (Figure 1).

On the other hand, among 56 gastric biopsy specimens containing *H. pylori*, 35 cases (62.5%) had the *cagA* gene (Figure 1). According to our results, the highest prevalence of the *cagA* gene belonged to patients with gastritis (20 of 28, *p*  <  0.05), compared to patients with peptic ulcer (12 of 21) and duodenum ulcer (3 of 7). Also, the frequency of the *cagA* gene in dental plaque specimens was 63.1% (12 of 19).

**Figure 2:**
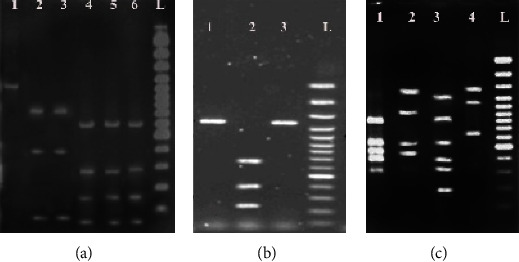
RFLP pattern of *vacA*, *cagA*, and *ureaAB* genes on agarose gel 2%. Part 2 (a): the RFLP patterns of *vacA*; lane 1: PCR product (1162 bp), lanes 2 and 3: the RFLP pattern of 3 bands (74, 392, 704 bp), lanes 4, 5, and 6: the RFLP pattern of 4 bands (50, 180, 293, 710 bp), lane L: size marker of 1000 bp; part 2 (b): the RFLP patterns of *cagA*; lanes 1 and 3: PCR product (1320 bp), lane 2: the RFLP pattern of 3 bands (254, 384, 712 bp), lane L: size marker of 100 bp; part 2 (c): the RFLP patterns of *ureAB*; lane 1: the RFLP pattern of 5 bands: (302, 373, 482, 550, 803 bp), lane 2: the RFLP pattern of 4 bands: (422, 512, 912, 1151 bp), lane 3: the RFLP pattern of 6 bands (182, 315, 381, 537, 845, 1213 bp); lane 4: the RFLP pattern of 3 bands: (650, 1152, 1284 bp), lane L: size marker of 100 bp.

Following enzymatic digestion of the *vacA* gene via HhaI and HphI (Figure 2(a)) and the *cagA* gene via HinfI (Figure 2(b)) on DNA extracted from gastric biopsy and dental plaque specimens, two RFLP patterns of 3 and 4 bands for the *vacA* gene and two RFLP patterns of 1 and 3 bands for the *cagA* gene were visually detected. The RFLP patterns of the *vacA* and *cagA* genes are shown in Table 2.

On the other hand, due to the enzymatic digestion of the *ureAB* gene (Figure 2(c)) by HaeIII, four RFLP patterns of 3, 4, 5, and 6 bands were visually recognized. The RFLP patterns of the *ureAB*, *vacA*, and *cagA* genes in the stomach samples are shown in Table 3. According to the data, we recognized 14 unique patterns.

According to Table 4, by combining the RFLP patterns of these three genes in the stomach and dental plaque specimens for the same case, 14 and 11 unique patterns were seen, respectively.

However, for all cases with a positive *H. pylori* diagnosis, a combined analysis of the RFLP patterns of these three genes was different in the dental plaque with the gastric biopsy samples for the same case. According to the data, there was no association between *H. pylori* colonization in the oral cavity and stomach for the same case (*p*  >  0.05) because the comparison of fingerprint patterns due to enzymatic digestion of three genes (*cagA*, *vacA*, and *ureAB*) showed that these patterns were different.

## 4. Discussion

In the past, the human stomach seemed to be the only reservoir of *Helicobacter pylori* colonization, until the identification of this bacterium was reported from some dental plaques, oral cavity, and saliva. However, the recovery of *H. pylori* from the oral cavity is a controversial issue. In addition, most attempts to isolate the bacterium from the oral cavity by culture failed [3]. Because *H. pylori* has a viable, noncultivable coccoid form, several investigators have proposed that *H. pylori* in this form may survive in the oral and is only detectable by nonculture methods [21, 22].

As an indirect test that can detect the presence of *H. pylori*, RUT has advantages over serology because it can only detect active *H. pylori* infection. To obtain a positive RUT result, there must be approximately 105 bacteria in the sample. Since, in our study, all patients with *H. pylori* colonization in the stomach and dental plaque (19 cases) had the same positive results for both RUT and PCR, therefore, it is concluded that the sensitivity of both tests is the same. In agreement with our study, some researchers also demonstrated that RUT sensitivity is higher or similar to PCR. Khalifehgholi et al. and Souod et al. have shown that the sensitivity of RUT and PCR was 95.6% and 93.5%, respectively [23, 24].

However, Chamanrokh et al. showed PCR as a more sensitive method rather than RUT for the detection of *H. pylori* [25]. Moreover, RUT is a specific method for the detection of *H. pylori* in gastric biopsy specimens, and in some cases, sensitivity and accuracy of 89.7% and 86.7% were shown, respectively, for this test in detecting *H. pylori* in dental plaque, but some researchers have questioned the validity of RUT for the diagnosis of *H. pylori* in oral specimens. Chamanrokh et al. believed that a negative RUT result does not necessarily exclude *H. pylori* infection and a positive-result RUT also does not confirm infection with this bacterium, because these results may be due to infection with other positive-urease bacteria [25]. Moreover, some studies indicate that false positive or negative results may occur. For example, the presence of other urease-positive organisms in the specimen can cause a false-positive result. Also, the use of antimicrobial drugs and proton pump inhibitors as well as the presence of intestinal metaplasia may result in false-negative results [26].

These controversial results can be explained by the fact that although *H. pylori* is the only urease-positive bacterium known in the gastric cavity, other urease-positive bacterial species, such as Streptococcus species, Haemophilus species, and Actinomyces species, also reside as part of the normal oral flora. However, according to some reports, only *H. pylori* can produce large amounts of urease for a short time, such as 20 min, while delayed urease-producing bacteria do not have positive results within one hour [11].

In the present study, we identified *H. pylori* by PCR method through the amplification of both urease and 16S rRNA genes in gastric biopsy and dental plaque specimens. *H. pylori* was found in the stomach of 52.8% (56/106) of the studied cases.

According to some reports, the frequency of *H. pylori* in dyspeptic patients in other regions of Iran using PCR was high and ranged from 48 to 85% [27–29]. This difference in rates can be explained according to the socio-economic status of population density in different regions of Iran, different methods in sample collection and processing, and primers used in PCR [6].

On the other hand, we found *H. pylori* simultaneously in dental plaques and gastric biopsies of 19 (17.9%) cases. Similar to our study, Eskandari et al. found *H. pylori* in 17.4% of cases in both the stomach and dental plaque samples simultaneously, in Iran [30]. Cellini et al. also showed that all the saliva and esophagus samples of 19 patients examined were positive by molecular analysis and suggested that these two sites may be considered reservoirs of *H. pylori* in humans, although they emphasize the need to use more sensitive techniques for *H. pylori* detection, especially in over-crowded sites [31]. Also, a simultaneous diagnosis of the same strain of *H. pylori* in both plaque and gastric mucosa was performed by Bharath et al. [32].

In contrast to our study, Momtaz et al. did not find any *H. pylori* in the dental plaques of patients with *H. pylori* gastritis, while they detected this bacterium in 77.6% of gastric biopsies, 10.7% of saliva, and 71.6% of stool samples [6]. These differences in the prevalence of *H. pylori* may be due to variations in sampling techniques, different methodologies, sets used in PCR, or some differences in the study population. In general, *H. pylori* colonization in the oral cavity is less than in the stomach. The low rate can be explained by two reasons: (1) suppressor effects of oral microbiota producing bacteriocin-like inhibitory proteins against *H. pylori* strains and (2) hiding *H. pylori* in yeast such as Candida spp. in the oral cavity [8]. Furthermore, unstable oral temperature due to eating and saliva as mechanical flushing may be a barrier to the long-term oral survival of *H. pylori* [5]. Although the performed investigations by many epidemiologists have shown a positive relationship between *H. pylori* in gastric infection and periodontal diseases [5, 33], many studies, however, could not confirm this association [34, 35]. Also, the study of Rossi-Aguiar et al. by evaluating the frequency of *cagA* and *vacA* genotypes of oral *H. pylori* in patients with functional dyspepsia showed that the oral cavity is not a reservoir for *H. pylori* in these patients [36].

In our study, the frequency of the *cagA* gene in the dental plaque and gastric biopsy specimens with *H. pylori*-positive results was 63.1% and 62.5%, respectively. The prevalence of *cagA*-positive *H. pylori* strains was different from one geographic region to another. A study in Austria showed that the prevalence of *cagA* in patients with gastric cancer and duodenal ulcers was 86% and 78%, respectively [37]. Also, the prevalence of this gene in Morocco, 42.3%, and in Egypt, 57.4%, has been reported [38, 39]. In addition, the prevalence of this gene was different in other regions of Iran, including 69% in Tehran [40], 92% in Shahrekord [24], and 69.7% in Tabriz [41].

In the present study, the number of *cagA*-positive *H. pylori* strains was higher in patients with gastritis than in patients with peptic ulcer and duodenum ulcer. However, Boukhris et al. and Eskandari et al. demonstrated a higher prevalence of this gene among patients with peptic ulcer (50%) and gastric cancer (85%), respectively [30, 38].

In this study, we found 14 and 11 unique patterns of RFLP in the stomach and dental plaque samples, respectively. In addition, to find the correlation between *H. pylori* colonization in dental plaque and stomach for the same case, the RFLP analyses of *vacA*, *cagA*, and *ureAB* genes were performed on the DNA extracted from the dental plaque and gastric biopsy samples. According to these analyses, different RFLP patterns were seen for each case; in other words, in each case, there were no identical RFLP patterns for both samples (dental plaque and gastric biopsy).

In summary, although finding the same strain in the mouth and stomach would support the role of the oral cavity as a reservoir for gastric *H. pylori*, however, more extensive studies are required to confirm the identity of closely related strains in the mouth and stomach [15].

## 5. Conclusions

It seems that in our study, there is no correlation between *H. pylori* colonization in dental plaque and the stomach. In other words, in this study, we could not consider dental plaque as a likely reservoir for reinfection of the gastric with *H. pylori*.

## Figures and Tables

**Table 1:** The sets of the used primers in this study.

Gene target	Primer 5′ to 3′	Amplicon size (bp)	Ref.
*16S rRNA*	F-GGAGGATGAAGGTTTTAGGAT	150	[17]
	R-TCGTTGCGGGACTTAACCCAA		

*vacA*	F-GCTTCTCTTACCACCAATGC	1162	[19]
	R-TGTCAGGGTTGTTCACCATG		

*cagA*	F-AGTAAGGAGAAACAATGA	1320	[19]
	R-AATAAGCCTTAGAGTCTTTTTGGAA		

*ureAB*	F- AGGAGAATGAGATGA	2420	[19]
	R-ACTTTATTGGCTGGT		

**Table 2:** The enzymatic RFLP patterns for *vacA* and *cagA* genes.

RFLP pattern	Gene		RFLP pattern	Gene	
	*vacA*			*cagA*	
	Dental *n* = 19(%)	Stomach *n* = 56(%)		Dental *n* = 12(%)	Stomach *n* = 35(%)
3 bands (74, 392, 704 bp)	9 (47.4)	36 (64.3)	1 band (%) (1320 bp)	3 (25)	10 (28.6)
4 bands (50, 180, 293, 710)	10 (52.6)	20 (35.7)	3 bands (%) (254, 384, 712 bp)	9 (75)	25 (71.4)
Total	19 (100)	56 (100)	Total	12 (100)	35 (100)

**Table 3:** The RFLP pattern of *ureAB*, *cagA*, and *vacA* genes on stomach specimens.

Patient number	Stomach		
	*ureAB*	*cagA*	*vacA*
1	6 bands	None	3 bands
2	5 bands	None	3 bands
3	5 bands	3 bands	4 bands
4	4 bands	3 bands	4 bands
5	5 bands	3 bands	4 bands
6	6 bands	3 bands	3 bands
7	5 bands	3 bands	4 bands
8	4 bands	1 band	4 bands
9	3 bands	3 bands	4 bands
10	6 bands	1 band	3 bands
11	6 bands	3 bands	3 bands
12	3 bands	3 bands	3 bands
13	6 bands	3 bands	3 bands
14	4 bands	1 band	3 bands
15	3 bands	3 bands	3 bands
16	6 bands	3 bands	4 bands
17	4 bands	3 bands	4 bands
18	6 bands	3 bands	3 bands
19	5 bands	None	3 bands
20	4 bands	None	4 bands
21	6 bands	None	3 bands
22	5 bands	None	3 bands
23	4 bands	3 bands	4 bands
24	5 bands	3 bands	4 bands
25	6 bands	None	3 bands
26	3 bands	3 bands	5 bands
27	4 bands	3 bands	4 bands
28	5 bands	None	3 bands
29	5 bands	3 bands	4 bands
30	6 bands	3 bands	3 bands
31	6 bands	None	3 bands
32	3 bands	3 bands	5 bands
33	6 bands	3 bands	3 bands
34	6 bands	None	3 bands
35	5 bands	3 bands	4 bands
36	4 bands	None	4 bands
37	3 bands	4 bands	3 bands
38	5 bands	None	3 bands
39	5 bands	3 bands	4 bands
40	3 bands	3 bands	5 bands
41	6 bands	None	3 bands
42	5 bands	3 bands	4 bands
43	4 bands	None	4 bands
44	3 bands	4 bands	3 bands
45	6 bands	None	3 bands
46	3 bands	3 bands	5 bands
47	4 bands	None	4 bands
48	6 bands	3 bands	3 bands
49	5 bands	None	3 bands
50	3 bands	4 bands	3 bands
51	4 bands	None	4 bands
52	5 bands	3 bands	4 bands
53	6 bands	None	3 bands
54	3 bands	3 bands	5 bands
55	4 bands	None	4 bands
56	4 bands	None	4 bands

**Table 4:** The combination of RFLP patterns of *ureAB*, *cagA*, and *vacA* genes on stomach and dental plaque specimens.

Patient number	Stomach			Dental plaque		
	*ureAB*	*cagA*	*vacA*	*ureAB*	*cagA*	*vacA*
1	6 bands	None	3 bands	3 bands	3 bands	3 bands
2	5 bands	None	3 bands	5 bands	3 bands	4 bands
3	5 bands	3 bands	4 bands	4 bands	None	3 bands
4	4 bands	3 bands	4 bands	6 bands	1 band	3 bands
5	5 bands	3 bands	4 bands	5 bands	None	3 bands
6	6 bands	3 bands	3 bands	4 bands	None	4 bands
7	5 bands	3 bands	4 bands	5 bands	None	3 bands
8	4 bands	1 band	4 bands	5 bands	3 bands	4 bands
9	3 bands	3 bands	4 bands	4 bands	3 bands	3 bands
10	6 bands	1 band	3 bands	5 bands	3 bands	3 bands
11	6 bands	3 bands	3 bands	4 bands	None	4 bands
12	3 bands	3 bands	3 bands	5 bands	None	4 bands
13	6 bands	3 bands	3 bands	5 bands	3 bands	3 bands
14	4 bands	1 band	3 bands	3 bands	3bands	3 bands
15	3 bands	3 bands	3 bands	4 bands	3 bands	3 bands
16	6 bands	3 bands	4 bands	5 bands	3 bands	4 bands
17	4 bands	3 bands	4 bands	5 bands	None	3 bands
18	6 bands	3 bands	3 bands	5 bands	None	4 bands
19	5 bands	None	3 bands	5 bands	None	4 bands

## Data Availability

All data generated or analyzed during this study are included in this article and are available upon reasonable request from the corresponding author.

## Additional Points

*Study Limitation*. One of the main limitations of our study was the lack of isolation of *H. pylori* by bacterial culture. Also, positive and negative controls have not been used in Figures 1 and 2(a), while they are needed in these Figures. The less number of female than male cases in this study can be considered as a limitation. *The presentation of the manuscript to Research Square*. Previously, an earlier and defective version of this manuscript was presented to Research Square, a company that offers multidisciplinary preprint and author services platform to help improve the research paper's communication.
